# Altered dietary behaviour during pregnancy impacts systemic metabolic phenotypes

**DOI:** 10.3389/fnut.2023.1230480

**Published:** 2023-12-04

**Authors:** Charlotte E. Rowley, Samantha Lodge, Siobhon Egan, Catherine Itsiopoulos, Claus T. Christophersen, Desiree Silva, Elizabeth Kicic-Starcevich, Therese A. O’Sullivan, Julien Wist, Jeremy Nicholson, Gary Frost, Elaine Holmes, Nina D’Vaz

**Affiliations:** ^1^Australian National Phenome Centre, and Centre for Computational and Systems Medicine, Health Futures Institute, Murdoch University, Perth, WA, Australia; ^2^Health and Biomedical Sciences, RMIT University, Melbourne, VIC, Australia; ^3^WA Human Microbiome Collaboration Centre, Curtin University, Bentley, WA, Australia; ^4^School of Medical and Health Sciences, Edith Cowan University, Joondalup, WA, Australia; ^5^Telethon Kids Institute, Perth Children’s Hospital, Nedlands, WA, Australia; ^6^Joondalup Health Campus, Joondalup, WA, Australia; ^7^Chemistry Department, Universidad del Valle, Cali, Colombia; ^8^Faculty of Medicine, Imperial College London, Institute of Global Health Innovation, London, United Kingdom; ^9^Section of Nutrition Department of Metabolism, Digestion and Reproduction, Faculty of Medicine, Imperial College London, London, United Kingdom

**Keywords:** Mediterranean diet, pregnancy, metabolic phenotype, inflammation, α-1-acid glycoprotein, hippurate, fibre

## Abstract

**Rationale:**

Evidence suggests consumption of a Mediterranean diet (MD) can positively impact both maternal and offspring health, potentially mediated by a beneficial effect on inflammatory pathways. We aimed to apply metabolic profiling of serum and urine samples to assess differences between women who were stratified into high and low alignment to a MD throughout pregnancy and investigate the relationship of the diet to inflammatory markers.

**Methods:**

From the ORIGINS cohort, 51 pregnant women were stratified for persistent high and low alignment to a MD, based on validated MD questionnaires. ^1^H Nuclear Magnetic Resonance (NMR) spectroscopy was used to investigate the urine and serum metabolite profiles of these women at 36 weeks of pregnancy. The relationship between diet, metabolite profile and inflammatory status was investigated.

**Results:**

There were clear differences in both the food choice and metabolic profiles of women who self-reported concordance to a high (HMDA) and low (LMDA) Mediterranean diet, indicating that alignment with the MD was associated with a specific metabolic phenotype during pregnancy. Reduced meat intake and higher vegetable intake in the HMDA group was supported by increased levels of urinary hippurate (*p* = 0.044) and lower creatine (*p* = 0.047) levels. Serum concentrations of the NMR spectroscopic inflammatory biomarkers GlycA (*p* = 0.020) and GlycB (*p* = 0.016) were significantly lower in the HDMA group and were negatively associated with serum acetate, histidine and isoleucine (*p* < 0.05) suggesting a greater level of plant-based nutrients in the diet. Serum branched chain and aromatic amino acids were positively associated with the HMDA group while both urinary and serum creatine, urine creatinine and dimethylamine were positively associated with the LMDA group.

**Conclusion:**

Metabolic phenotypes of pregnant women who had a high alignment with the MD were significantly different from pregnant women who had a poor alignment with the MD. The metabolite profiles aligned with reported food intake. Differences were most significant biomarkers of systemic inflammation and selected gut-microbial metabolites. This research expands our understanding of the mechanisms driving health outcomes during the perinatal period and provides additional biomarkers for investigation in pregnant women to assess potential health risks.

## 1 Introduction

Previous research into foetal programming has demonstrated that maternal lifestyle choices and exposures during the gestational period have a lasting impact on the offspring, in term of its association with chronic health conditions in later life, including neurodevelopment, diabetes and cardiovascular disease (1, 2).

The maternal diet is a modifiable lifestyle factor that can have a direct impact on the health of the developing foetus. Low quality diet can lead to poor growth rates and higher rates of birth complications and later life chronic health conditions (3, 4). An increasing interest in the impact of maternal nutrition on lifelong health of the offspring has led to a range of dietary studies either assessing the impact of single foods/nutrients or evaluating dietary patterns during pregnancy. For example, consumption of fish during pregnancy has been associated with benefits in neurocognitive development in the offspring (5), while an association between lower birth weight and diets that are high in processed foods, saturated fats and sugars has been found (6). As nutrients are not consumed in isolation, and the impact of the food matrix cannot be understated, studying the impact of whole diet is more appropriate to community-based interpretation of research outcomes.

The Mediterranean diet is one of the most well-studied diets and numerous investigations have assessed the impact of consuming this diet during pregnancy on birth or childhood development outcomes. It is generally accepted that consumption of a Mediterranean diet during pregnancy is associated with benefits to both the mother and offspring, particularly with reference to systemic inflammation (7, 8). Adherence to the Mediterranean diet during pregnancy has been linked to reduced likelihood of gestational diabetes (9), reduced lipid oxidation and DNA damage in mothers (10), and a reduction in preterm births (6). In the offspring, maternal adherence to consumption of a Mediterranean diet during pregnancy was associated with lower rates of overweight and reduced body fat percentage, including waist circumference (11), improved cognitive and executive function in offspring (9), reduced incidence of atopic diseases (12) beneficial differences in DNA methylation and microRNAs (13) and a lower offspring systolic and diastolic blood pressure (14). Some studies have assessed alignment with a MD, including during pregnancy. Previous studies investigating the impact of the MD during pregnancy have shown that although increasing MD alignment was not associated with a significant change in BMI, it was associated with lower pregnancy weight gain (15, 16). Other studies have demonstrated that greater adherence to the Mediterranean dietary pattern during the first trimester of pregnancy may be favourably associated with communication abilities at 6-month aged infants (17) as well as lower risk of adverse pregnancy outcomes, with evidence of a dose–response association (18). However, despite the general consensus of the benefits of a maternal Mediterranean diet, several studies have not found associations between maternal diet and infant health (19), highlighting the challenges in assessing dietary impact on foetal programming.

One potential mechanism of foetal programming is through maternal systemic inflammation levels. During pregnancy inflammation naturally increases from the time of blastocyst implantation and escalates during parturition (20) and pregnancies with complications such as preeclampsia and gestational diabetes show raised levels of the inflammatory marker C-Reactive Protein (CRP) compared to pregnancies without complications (21). Plasma acute phase glycoprotein glycans (GlycA and GlycB), arising from N-acetylglucosamine/galactosamine and neuraminic (or sialic) acid chains, can be measured using ^1^H nuclear magnetic resonance (NMR) spectroscopy (22, 23) and have been shown to be more reliable than high sensitivity CRP (24). These signals are associated with acute phase proteins such as ɑ-1-acid glycoprotein, transferrin, haptoglobin, serotransferrin and ɑ-1-antitrypsin (25). Studies in other pregnancy cohorts have shown a direct association between GlycA and BMI (26) and gestational diabetes (27). It is unknown whether reducing inflammation during pregnancy can result in improved pregnancy outcomes.

Diets which are high in saturated fats, meats, processed and sugary foods, as well as being low in whole grains, fruits and vegetables, and healthy fats, tend to be associated with systemic inflammation (28, 29). Conversely, plant-based diets, such as the Mediterranean Diet (MD), high in whole grains and healthy fats such as avocado and olive oil, tend to have lower levels of inflammatory metabolites (29). This style of eating has been adopted as an anti-inflammatory dietary tool in general dietary practice (30). However, the mechanisms by which adherence to a Mediterranean diet achieves this anti-inflammatory effect are not yet fully understood.

Metabolic phenotyping, using high resolution spectroscopic technologies, has proven to be a useful tool in dietary assessment (31–33). It has been used to assess compliance through dietary metabolite biomarkers and to elucidate metabolic profiles associated with consumption of a Mediterranean diet, either as a lifestyle choice (34) or as a tool for reducing cardiometabolic disease risk (35). Here, we apply ^1^H nuclear magnetic resonance (NMR) spectroscopy to assess differences between women who were stratified into high and low alignment with a Mediterranean diet throughout their pregnancy and analyse the spectral profiles with respect to foods and food groups identified from Food Frequency Questionnaires (FFQ’s). We anticipate significant metabolic differences between those in a high alignment and low alignment with the MD, with those highly aligned to the MD exhibiting lower levels of inflammatory biomarkers.

## 2 Materials and methods

### 2.1 Patient enrolment and sample collection

**Figure 1:**
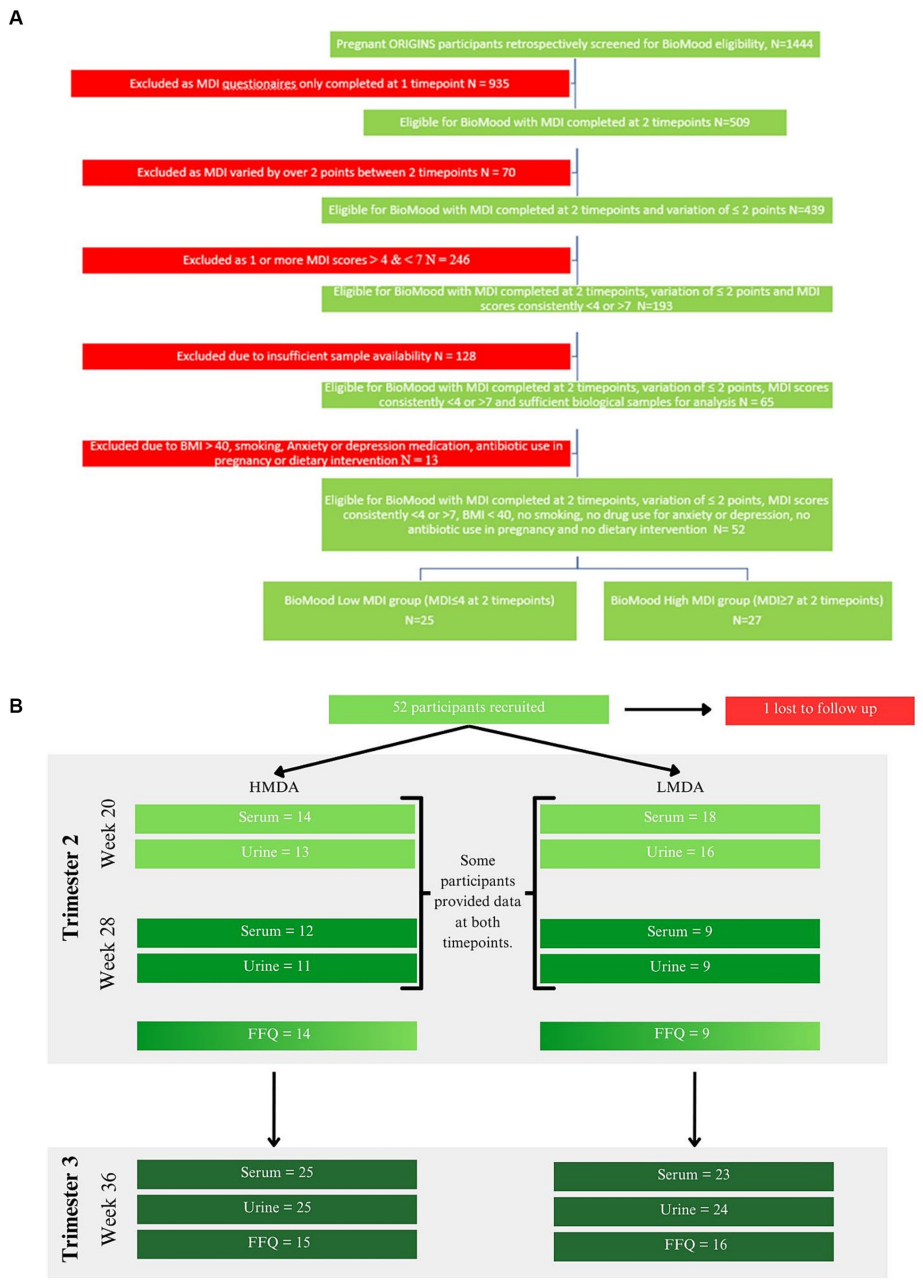
**(A)** Study selection criteria and collection protocol, **(B)** food data and sample collection cohorts for each hospital visit timepoint.

The BIOMOOD study was established retrospectively as a subsidiary of the ORIGINS project, a longitudinal study of family health outcomes, commencing during pregnancy. The study selection criteria and collection protocol are provided as a schematic (Figure 1A) including food data and sample collection details (Figure 1B). The study was approved by the ORIGINS Scientific Committee and Project Management Group (Application ID: ND01905) and Ramsay Health Care Human Research Ethics Committee (Protocol number: ND01905). Sample collection methods have been described in detail elsewhere (36) but include the collection of dietary information socio-demographic and anthropomeric data, and urine and serum samples. Briefly, pregnant women who attended Joondalup Health Campus in Australia for antenatal care were invited to participate in the ORIGINS project at approximately 18 weeks gestation. Initial samples were collected at either 20 weeks or 28 weeks of gestation, dependent on their presentation to the antenatal clinic, and then again at 36 weeks. Participants completed several questionnaires, including a validated Mediterranean Diet Questionnaire (MDQ) (37) and FFQ. The FFQ used was the Australian Eating Survey (38). The MDQ is a validated 14-point assessment which focusses on olive oil use, wine intake, legume consumption, fish intake, and the consumption of chicken over red meats, and has previously been used to assess diet in pregnant women (15). Since alcohol intake is discouraged during pregnancy, this item was excluded from the MDQ scale (see Supplementary Table S1). Items were scored simply as meeting requirement, or not meeting requirement, and therefore the range of potential scores for the MDQ is between 0 and 13. Only participants who completed at least two MDI assessments during their pregnancy were included in the current study.

Participants were assigned to groups dependent on their MDI scores at week 20 and 28 and were stratified according to Ashwin et al., whereby low alignment (LMDA) was considered a score between 0 and 4, medium alignment between 5 and 7 and high alignment (HMDA) between 8 and 13 (39). The inclusion criteria were such that no individual was included if the MDI score deviated more than 2 points between the collections, therefore we accepted participants where at least one of the MDI values met the criteria for low or high alignment with the Mediterranean Diet. Participants were also excluded if they did not have biological samples available for analysis, were on antibiotics at any time during their pregnancy, reported using antidepressants or anti-anxiety medication or had a pre-pregnancy BMI of over 40 kg/m^2^. A total of 52 women were determined to be eligible for the BIOMOOD study, with one participant excluded due to dropout, leaving 26 participants assigned to the HMDA group, and 25 assigned to the LMDA group (demographics provided in Table 1). Participants provided urine and blood (serum collected into lithium heparin tubes). Samples were aliquoted to allow for multiple analyses and stored at −80°C at the Telethon Kids Institute in Western Australia as part of their ongoing BioBank. A serum and urine aliquot for each participant was transferred frozen to the Australian National Phenome Centre, where they were again stored at −80°C prior to analysis.

**Table 1:** Demographic information of participants in the BIOMOOD study.

	Low Mediterranean diet alignment (*n* = 25)	High Mediterranean diet alignment (*n* = 26)	*p*-value
Age in years (SD)	31.28 (3.75)	32.92 (3,93)	0.133^**^
Pre-pregnancy weight, kg (SD)	74.40 (11.57)	70.54 (15.13)	0.312^**^
Pre-pregnancy BMI, kg/m^2^ (SD)*	28.00 (4.17)	25.28 (5.43)	0.056^**^
Parity (%)			0.331^***^
*0*	20.00 (80.00%)	16.00 (61.54%)	
*1*	4.00 (16.00%)	7.00 (26.92%)	
*2*	1.00 (4.00%)	3.00 (11.54%)	
Education (%)			0.689^***^
*Year 10*	1.00 (4.00%)	0.00 (0.00%)	
*Year 12*	4.00 (16.00%)	1.00 (3.85%)	
*Trade*	2.00 (8.00%)	3.00 (11.54%)	
*Bachelor*	10.00 (40.00%)	12.00 (46.15%)	
*Postgrad*	6.00 (24.00%)	8.00 (30.77%)	
*Other*	2.00 (8.00%)	2.00 (7.69%)	
Employment (%)			0.629^***^
*Not seeking work*	0.00 (0.00%)	1.00 (3.85%)	
*Home duties*	3.00 (12.00%)	4.00 (15.38%)	
*Periodic work*	0.00 (0.00%)	1.00 (3.85%)	
*Casual work*	1.00 (4.00%)	2.00 (7.69%)	
*Part time work*	7.00 (28.00%)	3.00 (11.54%)	
*Full time work*	14.00 (56.00%)	14.00 (53.85%)	
*Other*	0.00 (0.00%)	1.00 (3.85%)	

Values reported as mean with (standard deviation) or number of participants (percentage). * Missing BMI data for two participants (one in HMDA and one in LDMA). *p*-values reported for ^**^ two-sided independent *t*-test and ^***^ two-sided Fisher’s test.

### 2.2 Sample preparation

In preparation for analysis, the serum samples were thawed at room temperature for 30 min, before undergoing centrifugation at 13,000 g for 10 min at 4°C. Samples were prepared in SampleJet™ NMR tubes of 5 mm outer diameter using a standard preparation method (40) of 300 μL of serum mixed with 300 μL phosphate buffer (75 mM Na_2_HPO_4_, 2 mM NaN_3_, 4.6 mM sodium trimethylsilyl propionate-[2,2,3,3-^2^H_4_] (TSP) in H_2_O/D_2_O 4:1, pH 7.4 ± 0.1).

Similarly, urine was thawed at room temperature for 30 min, before undergoing centrifugation at 13,000 g for 10 min at 4°C. Samples were prepared in SampleJet™ NMR tubes of 5 mm outer diameter using a standard preparation method (40) of 540 μL of urine mixed with 60 μL phosphate buffer (1.5 mM KH_2_PO_4_, 2 mM NaN_3_, 0.1% TSP, pH 7.4 ± 0.1).

#### 2.2.1 Acquisition and preprocessing of ^1^H NMR serum spectra

A 600 MHz Bruker Avance III HD spectrometer equipped with a 5 mm BBI probe, utilising a Bruker SampleJet™ robot cooling system set to 5°C, was used to perform NMR spectroscopic analyses. Prior to the analysis, a full quantitative calibration was completed using a previously described protocol (40). Three experiments were performed in automation mode for each serum sample, taking a total experimental acquisition time of 12 min. These experiments were a 1D experiment with solvent pre-saturation (32 scans, 98 K data points, spectral width of 30 ppm), a spin-echo experiment (32 scans, 74 K data points, spectral width of 20 ppm) and a diffusion-relaxation edited experiment (JEDI-PGPE: 64 scans, 98 K data points, spectral width of 30 ppm) (41). For both urine and serum, the standard 1D experiment was acquired using the Bruker In Vitro Diagnostics Research (IVDr) protocol to allow for quantification. All data were processed in automation using Bruker Topspin 3.6.2 and ICON NMR to achieve phase correction and calibration to TSP (δ 0). Bruker IVDr Quantification in Serum/Serum B.I.Quant-PS provided data on 25 low molecular weight metabolite concentrations (acetic acid, acetoacetic acid, acetone, alanine, citric acid, creatine, creatinine, formic acid, glucose, glutamic acid, glutamine, glycine, histidine, D-3-hydroxybutyric acid, isoleucine, lactic acid, leucine, lysine, N,N-dimethylglycine, methionine, phenylalanine, pyruvic acid, trimethylamine-*N*-oxide, tyrosine, and valine) and B.I.LISA provided 112 lipoprotein parameters. For serum a CPMG spin-echo experiment (42), which performs differential T2 relaxation to filter the spectrum, was used to remove signals from large molecules with fast relaxing protons and the JEDI-PGPE employed diffusion and relaxation spectral editing to enhance the signals from the inflammatory panel (GlycA, GlycB, and the supramolecular phospholipid composite (SPC) peak) (41).

Upon completion of spectral acquisition, processing using in house developed R scripts included: division of spectral data points for each of the three NMR experiments (standard 1D with water suppression, spin echo, JEDI-PGPE) by the eretic factor (electronic quantitation standard) (42). Both the standard 1D and CPMG NMR spectral datasets were calibrated to the α-anomeric proton signal of glucose at δ 5.23, while no calibration was necessary for the JEDI experiments. Spectral regions corresponding to the residual water resonance signal (δ 4.60–4.85) or predominantly noise (δ < 0.5 and δ > 9.5) were excluded from analyses and the spin-echo spectrum was corrected for baseline distortions using an asymmetric least squares routine using the R package metabom8 (version 1.0.0), available from GitHub.^1^ To estimate the signal intensities of GlycA and GlycB peaks, spectral regions were integrated (GlycA: δ 2.03; GlycB: δ 2.07) from the JEDI-PGPE spectra. The GlycA signal (δ 2.03) is a composite of *N*-acetyl signals from five proteins: α-1-acid glycoprotein (major component), α-1-antitrypsin, α-1-antichymotrypsin, haptoglobin, and transferrin. The GlycB acetyl signal (δ 2.07) arises from glycoprotein N-acetylneuraminidino groups (41). The region containing the supramolecular phospholipids composite peak (SPC) was also integrated from the JEDI-PGPE spectra (δ 3.20–3.30). This composite peak was further subdivided into 3 markers, SPC1 (δ 3.2–3.236) corresponding predominantly to small HDL (HDL4) phospholipids, SPC2 (δ 3.236–3.252) corresponding to larger HDL phospholipid particles (HDL1-3) and SPC3 (δ 3.252–3.3) corresponding to LDL phospholipids (43). In addition, the sum of Glyc (Glyc A + Glyc B) and SPC (SPC1, SPC2, and SPC3) ratios were calculated of Glyc A /Glyc and SPC (total) / Glyc (total).

#### 2.2.2 ^1^H NMR spectroscopy data acquisition and processing parameters for urine samples

The same spectroscopic platform (see Section 2.2.1) was used to record spectra of urine samples. Prior to analysis, a full quantitative calibration was completed using a previously described protocol (40). A standard 1D experiment, acquired using the Bruker In Vitro Diagnostics Research (IVDr) methods, was performed in automation mode for each urine sample using solvent pre-saturation, taking a total experimental acquisition time of 4 min; 32 scans; 65 K data points; spectral width of 20 Hz. All spectra were processed in automation using Bruker Topspin 3.6.2 and ICON NMR to achieve phase and baseline correction, and calibration to TSP (δ = 0).

Further processing was undertaken using in house developed R scripts: For each spectrum, the regions corresponding to the residual water resonance signal (δ 4.75–4.85) or predominantly noise (δ < 0.5 and δ > 9.5) were excluded and each data point was divided by the eretic factor to obtain quantitative values. The concentrations of urinary metabolites associated with alignment to the Mediterranean Diet were obtained either from the Bruker IVDr Quantification procedure B.I.Quant-UR b or an in-house curve resolution algorithm in the cases where the metabolite was not found in the B.I.Quant-UR b data. Combined Multi-block Principal components Analysis with Statistical Spectroscopy (COMPASS) (44) was used to estimate the concentrations of 4-cresol sulfate (δ = 2.35) and phenylacetylglutamine (PAG) (δ = 7.36–7.45) and expand the B.I.Quant-UR b panel.

### 2.3 Statistical analysis

All computation and data visualisation were performed using R (4.1.0) and RStudio IDE. Comparison of cohort demographics between the two diet groups was assessed using two-sided independent *t*-test (continuous variables) or two-sided Fisher’s test (categorical variables) (Table 1). Multivariate modelling was achieved using the open-source R package metabom8 (release 1.0.0), available from GitHub (see text footnote 1) and Combined Multi-block Principal components Analysis with Statistical Spectroscopy (COMPASS) was achieved using the scripts openly available (41). Principal Component Analysis (PCA) and Orthogonal projection to latent structures-discriminant analysis (OPLS-DA) (45) were used to model variance in the data and to extract discriminating features between the two diet groups. Metabolite identification was undertaken using statistical correlation spectroscopy (STOCSY) (46), by comparison with reference standards found either in house or in databases including HMDB (47) and by using 2-dimensional NMR experiments including COSY, TOCSY, HMQC and J-Resolved carried out on representative samples to confirm tentative assignments from databases (48).

Based on the OPLS-DA model, the concentrations of nine low molecular weight metabolites were used for further univariate analysis (arginine, citric acid, creatine, creatinine, dimethylamine, glycine, hippuric acid, *N,N*-dimethylglycine, and trigonelline).

**Figure 2:**
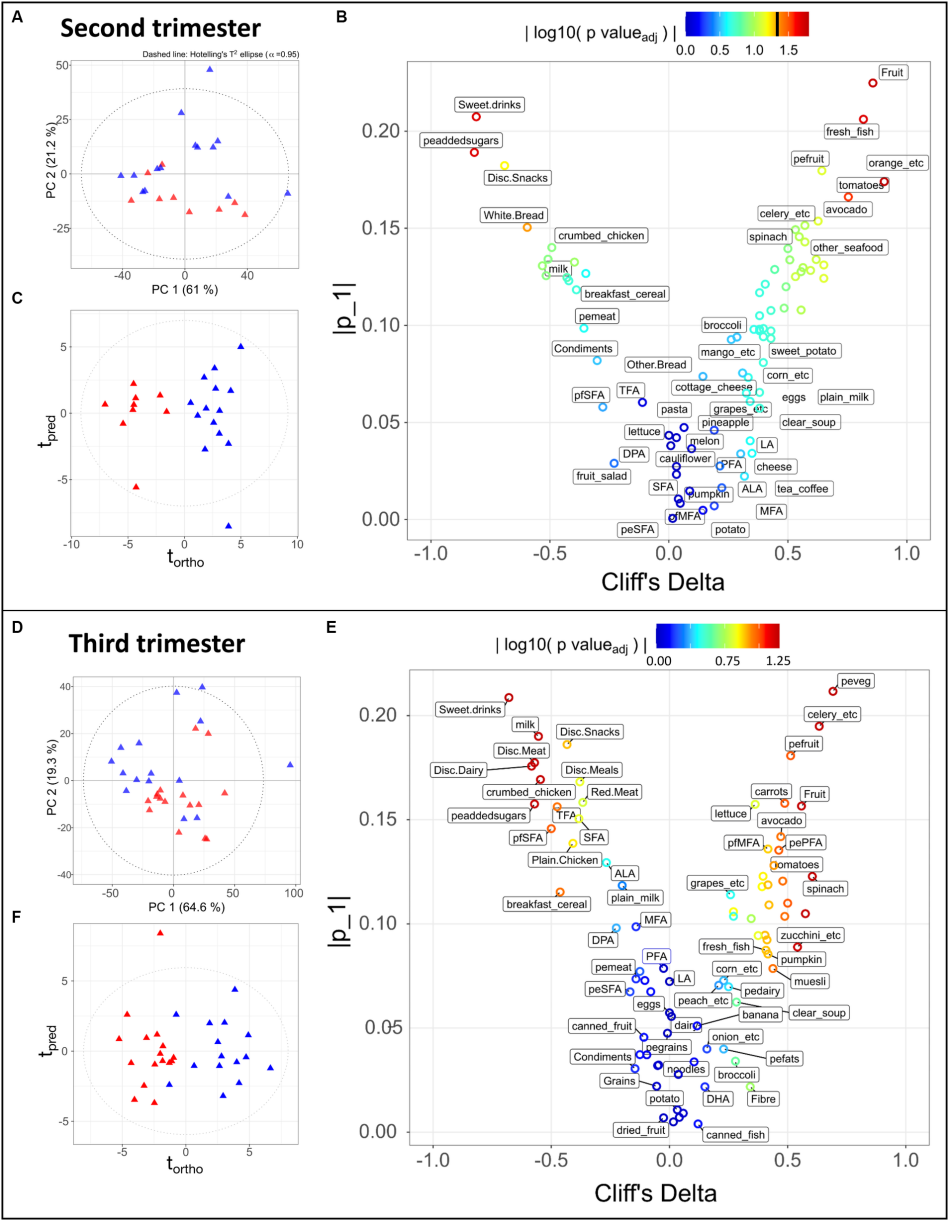
Statistical analysis of 83 dietary components obtained during the second trimester **(A–C)** for 9 LMDA, 14 HMDA participants; and third trimester **(D–F)** for 16 LMDA, 15 HMDA participants. **(A,D)** PCA scores plot of dietary components, classified by dietary adherence (low-red triangles or high-blue triangles), **(B,E)** OPLS-DA scores plots of dietary components, classified by dietary adherence (low-red triangles or high-blue triangles), **(C,F)** Eruption plots of dietary components, demonstrating the driving components in the model differentiating the two dietary adherence groups. For figures **(B,E)**, the color represents the magnitude of the reconstructed loadings, the vertical axis represents the univariate statistics, while the Cliff’s delta is represented on the *x*-axis. This latter is used as non-parametric estimator of the effect size, with values outside of the ±1 interval representing samples with no overlap. pefats, percentage of energy from fats; DHA, docosahexaenoic acid; EPA, eicosapentaenoic acid; DPA, docosapentaenoic acid; ALA, alpha-linolenic acid; LA, linoleic acid; MFA, monounsaturated fatty acids; peMFA, percentge of energy from monounsaturated fatty acids; peaddedsugars, percentage of energy from added sugars; PFA, polyunsturated fatty acids; pePFA, percentage of energy from polyunsaturated fatty acids; pfpolyfats, percentage of fat from polyunsaturated fat; pfSFA, percentage of fats coming from saturated fatty acids; peSFA, percentage of energy from saturated fatty acids; SFA, saturate fatty acids; TFA, transaturated fatty acids; pemeat, percentage of energy from meat; pefruit, precentage of energy from fruit; peveg, percentage of energy from vegetables; pedairy, percentage of energy from dairy; pegrains, percentage of energy from grains; Disc, discretionary.

Dietary data obtained during the second trimester were available for 23 participants (9 LMDA and 14 HMDA). At the third trimester, dietary data were recorded for 31 participants (16 LMDA and 15 HMDA). Seven participants in each diet group had data available at both timepoints. PCA using Pareto scaling, eruption plots and OPLS was performed with 83 dietary components at both time points. For multivariate analysis dietary components were grouped together into related food groups (see Supplementary Table S2 for full list of dietary items). A final set of 40 variables were identified, which included the MDI score obtained at weeks 20 or 28 and at week 36, the amount of fibre expressed as grams per 1,000 kJ, 24 food frequency variables, ten variables related to percentage of energy, and three variables related to percentage of fat intake (see Supplementary Table S3 for further details). Spearman pairwise rank correlation analysis was used to investigate relationships between dietary components as well as between dietary components and metabolites. Whereas all participants attended the visit at 36 weeks in trimester 3, with the exception of a few participants, attendance in the second trimester was either at week 20 or 28. While data from both available trimesters were investigated, associations between diet and metabolic profiles were drawn solely from trimester three data. However, for the FFQ data we explored the consistency of the reported data across both trimesters to ascertain the reproducibility of dietary reporting (Figure 2).

Post-partum outcomes were not assessed as part of the BIOMOOD study.

## 3 Results

### 3.1 Cohort characteristics

In the group of 51 pregnant women enrolled in the study, the mean age was 32.12 (SD 3.89) years, with an average pre-pregnancy BMI of 26.62 kg/m^2^ (SD 4.99 kg/m^2^). No significant differences were noted between the demographics of the two groups (Table 1).

### 3.2 Dietary analysis

Of the 51 women for whom urine and serum samples were available, 41 provided food frequency questionnaires (FFQ’s) for at least one timepoint, from which the estimated intake of total energy and nutrients was obtained. Nutrients were expressed as a percentage of total energy and dietary fibre per 1,000 kJ. As these participants either provided their initial biological sample at week 20 or week 28, the sample cohort for these time points was significantly reduced for both groups. As such, we decided to focus the nutritional analysis on the week 36 timepoint, for which 31 participants had provided dietary data. Figure 2 demonstrates the dietary differences between the groups in the second and third trimesters, with further details in Supplementary Tables S4A,B. As the dietary patterns remained consistent between the two time points, this supported our decision to use the more complete dietary data from the week 36 timepoint.

Principal Component Analysis (PCA) is an unsupervised multivariate method that finds the largest degrees of variance in a dataset. It is therefore suitable for evaluating the overall data quality, identifying outliers or of unexpected sources of variance, and confirming the presence of trends in the data, i.e., separation between groups. Such a trend can be observed in the PCA scores depicted in Figures 2A,D, where the HMDA and LMDA groups naturally separate. One limitation of PCA is that it cannot be used to model a specific source of variance, such as the one of interest (diet group) in our experimental design. In contrast, Projections to Latent Structures (PLS) is a family of supervised methods that require prior knowledge of categories or groups to model a single degree of variance. Once a model is trained and validated its scores will reflect the selected variance (predicted) on the x dimension, while unrelated source of variance (orthogonal) will be displayed on the y-axis. Therefore, Orthogonal Projection to Latent Structures Discriminant Analysis (OPLS-DA) models were calculated for alignment to the Mediterranean diet using the participants with available FFQ data. The resulting scores demonstrated clear separation between the two dietary alignment groups (Figures 2C,F) (Trimester 2, *R*^2^X = 0.21, CV-AUROC = 0.96; Trimester 3, *R*^2^X = 0.17, CV-AUROC = 0.82). The model loadings were used to identify the variables that contributed most to explaining the score for each individual participant. An eruption plot of the FFQ’s or “nutrigram” was created from the loadings to contrast univariate non-parametric pair testing and Cliff’s deltas (Figures 2B,E). The nutrigram shows that, as expected, participants in the HMDA group were consuming the anticipated foods based on a typical Mediterranean diet profile (Figures 2C,F). The variables with a Cliff’s delta statistic −0.4 > cd > 0.4 are listed for both trimesters in Supplementary Tables S5A,B, and are color coded according to food group. A significantly higher intake of vegetables was evident for the HMDA group overall, especially green vegetables such as celery, cabbage and spinach, and higher intakes of fresh fish and nuts. Comparatively, the LMDA group demonstrated a diet driven by highly processed foods such as muffins and takeaway foods, and especially soft drink consumption (Figure 2E and Supplementary Tables S4A,B). Dietary elements such as overall dairy intake, canned and dried fruits and eggs did not contribute to the difference between the two dietary groups. These dietary patterns are consistent with the anticipated dietary intakes for people based on their level of alignment with the Mediterranean diet and the patterns remained relatively consistent between trimester 2 and trimester 3, supporting the analysis, which demonstrated minimal dietary change over the course of gestation.

Correlation maps of the dietary variables based on all participants (Supplementary Figure S1) showed expected relationships between food components. For example, added sugars expressed as percentage of energy intake significantly correlated with sweet drinks, discretionary snacks (such as soft drink, juice, cordials, potato chips, jelly, and chocolate etc.) and inversely correlated with fresh fruits; fresh vegetables were correlated with vegetables and percentage energy and starch; monounsaturated dietary fats correlated with saturated dietary fats; and nuts and legumes correlated with vegetables.

### 3.3 Alignment to Mediterranean diet is reflected in the serum NMR profiles

**Figure 3:**
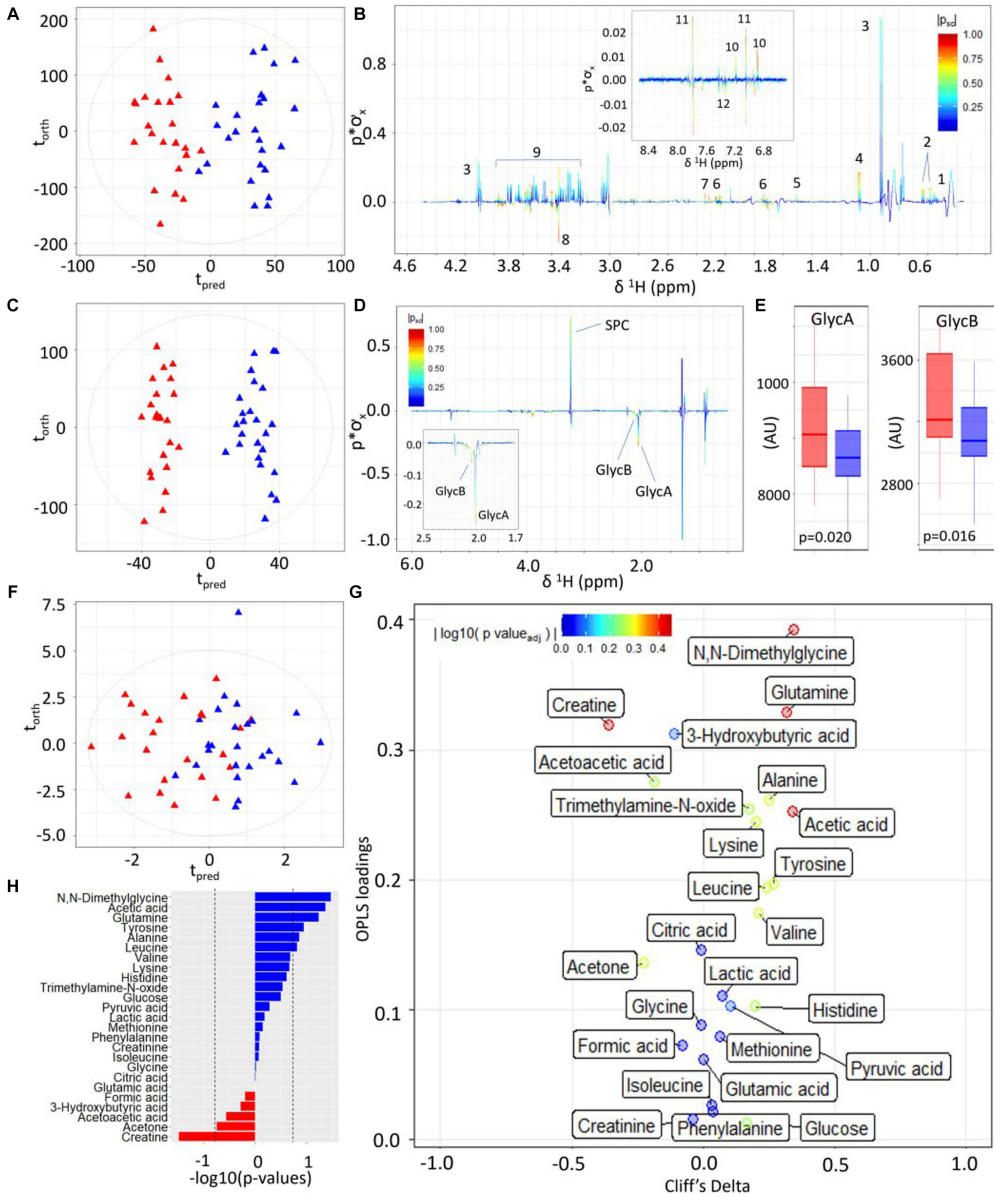
Statistical comparison of third-trimester serum metabolite profiles of participants with high versus low alignment to the Mediterranean Diet showing higher concentrations of inflammatory-associated metabolites and lower concentrations of amino acids and ketone bodies. **(A)** OPLS-DA scores plot of 1D ^1^H CPMG NMR profiles of serum samples taken at trimester three, classified by HMDA (blue triangles) or LMDA groupings (red triangles) (*R*^2^X = 0.04, CV-AUROC = 0.68), **(B)** ^1^H NMR backscaled coefficients plot from OPLS-DA model based on ^1^H CPMG NMR showing significant metabolite peaks at trimester 3, with insert of aromatic region indicating a significant increase of hippurate in the HMDA group. Key: 1-leucine, 2-valine, 3-lactate, 4-alanine, 5-acetic acid, 6-glutamine, 7-citrate, 8-glycine, 9-glucose, 10-tyrosine, 11-histidine, 12-phenylalanine. **(C)** OPLS-DA scores plot of JEDI-PGPE NMR data (*R*^2^X = 0.03, CV-AUROC = 0.68), **(D)** backscaled coefficients plot for JEDI-PGPE model, **(E)** box plots of GlycA and GlycB concentrations, **(F)** OPLS-DA scores plot of small molecule metabolites (*R*^2^X = 0.09, CV-AUROC = 0.63), **(G)** Eruption plot of quantified small molecule concentrations, **(H)** −log10 *p*-values of all the small molecules. Blue bars represent metabolites that are increased in the HMDA group, while those in red are increased in the LMDA group. SPC, supramolecular phospholipid composite; GlycA, acute phase glycoprotein glycan A; GlycB, acute phase glycoprotein glycan B.

Serum samples taken at 36 weeks were available for 48 participants (23 LMDA, 25 HMDA). The CPMG/spin-echo serum ^1^H NMR profiles of the HMDA and LMDA group demonstrated a number of systematic differences in the OPLS-DA models (Figure 3A), which was driven by significantly higher concentrations of the amino acids: valine, alanine, glutamine and tyrosine, as well as increased levels of acetone and acetate for the HMDA group (Figure 3B). Conversely glycine was present in higher concentrations in the LMDA group. Histidine and methylhistidines also differed between groups but this was not apparent from the OPLS-DA loadings due to the high degree of chemical shift. Standard comparison of the spectra showed these histidines to be present in relatively higher concentrations in the HMDA group and histidine was correlated with foods associated with HMDA in the metabolite-food plot (see Supplementary Figure S2).

Similarly, the two dietary groups were completely differentiated in the models using the JEDI-PGPE spectra that enhances the contribution from the *N*-acetylated glycoproteins and Supramolecular Phospholipid Composite Peaks (SPC) (CV-AUROC = 0.68) (Figure 3C). These differences were almost entirely driven by the stronger presence of GlycA (*p*-value = 0.020) and GlycB (Figures 3D,E; *p*-value = 0.016) in the LMDA group. The composite supramolecular phosphocholine peak SPC (δ 3.20–3.30), was not significantly different between the two diets but showed a trend towards higher concentrations in the HMDA score.

Systematic differences between the two dietary alignment groups were also visible in the OPLS-DA model calculated using the quantified metabolites extracted using the IVDr procedure (Figure 3F). Here the main differences in metabolite composition were visualised using an eruption plot (Figure 3G). The HMDA group was characterised by *N,N*–dimethylglycine, glutamine and acetate for the HMDA group, whereas creatine was significantly higher in the LMDA group. Although the quantified branched chain amino acids were not significantly different between groups, there was a trend towards higher levels in the HMDA group, matching the results established using the multivariate OPLS-DA analysis of the spectral profiles. Similarly, alanine and tyrosine were associated with the HMDA group but did not achieve significance in the univariate analysis after adjusting for multiple testing.

### 3.4 Differential urine ^1^H NMR profiles between HMDA and LMDA groups

**Figure 4:**
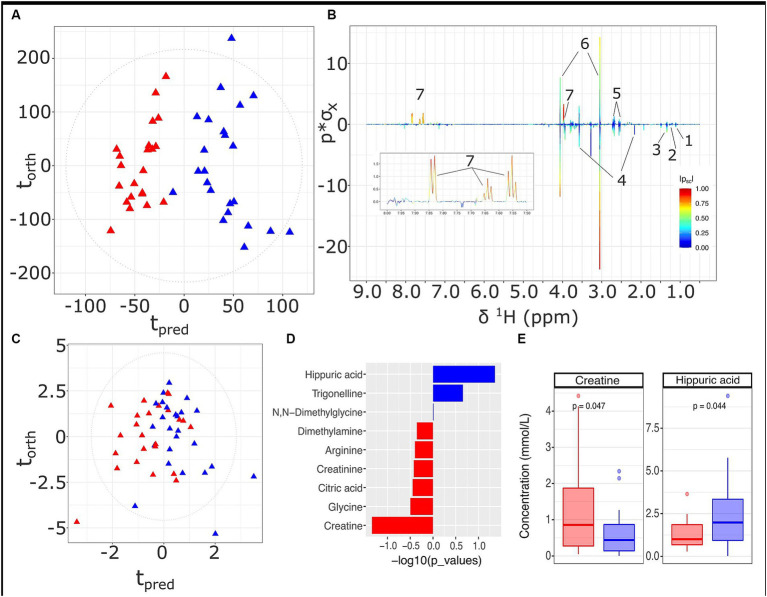
Urine metabolite analysis for samples obtained at 36 weeks, LMDA (*n* = 22) in red and HMDA (*n* = 23) shown in blue. **(A)** OPLS-DA scores plot of standard 1D ^1^H NMR profiles of urine samples taken at trimester three, classified by HMDA or LMDA groupings (*R*^2^X = 0.05,CV-AUROC = 0.84), **(B)** ^1^H NMR backscaled coefficients plot showing significant metabolite peaks of 1D OPLS-DA at trimester 3, with insert of aromatic region to highlight the increase of hippuric acid in the HMDA group; **(C)** OPLS-DA scores plot of small molecules (*R*^2^X = 0.31, CV-AUROC = 0.72). **(D)** −log10 *p*-values of the nine small molecules. Metabolites denoted by blue bars are increased in the HMDA group, while those in red are increased in the LMDA group. **(E)** Boxplot of creatine and Hippurate, with statistical significance in differentiating the LMDA and HMDA groups. Key: 1. 0.4-Deoxyerythronic acid, 2. 4-Deoxythreonic acid, 3. Lactate, 4. p-Cresol sulfate, 5. Citrate, 6. Creatinine, 7. Hippurate.

A differential urinary signature was found for the HMDA versus the LMDA group based on the OPLS-DA model scores and loadings plot (Figures 4A,B respectively) built from the ^1^H NMR spectra of urine samples obtained at 36 weeks of gestation (23 LMDA, 24 HMDA) yielding a CV-AUROC of 0.84. The loadings coefficients for the model (Figure 4B) indicated that hippurate dominated the separation between the two dietary groups, being present in significantly higher concentrations in the urine of those individuals who were highly adherent to the Mediterranean diet. Analysis of the quantified low molecular weight metabolites provided by the IVDr method (Figure 4C,D and Supplementary Figure S4) also supported increased hippurate in the HMDA, as well as trigonelline. Like the serum profiles, creatine was again noted as being present in significantly higher concentrations in those who were least adherent to the Mediterranean diet (Figure 4E). Sarcosine, alanine, and citrate were additionally identified as being present in higher concentrations in the LMDA group, although the difference was not significant due to a high degree of inter-individual variation (Supplementary Figures S5, S6 and Figures 4C,E,F).

### 3.5 Metabolite and diet-metabolite correlations underlying the differences in alignment with a Mediterranean diet pattern

**Figure 5:**
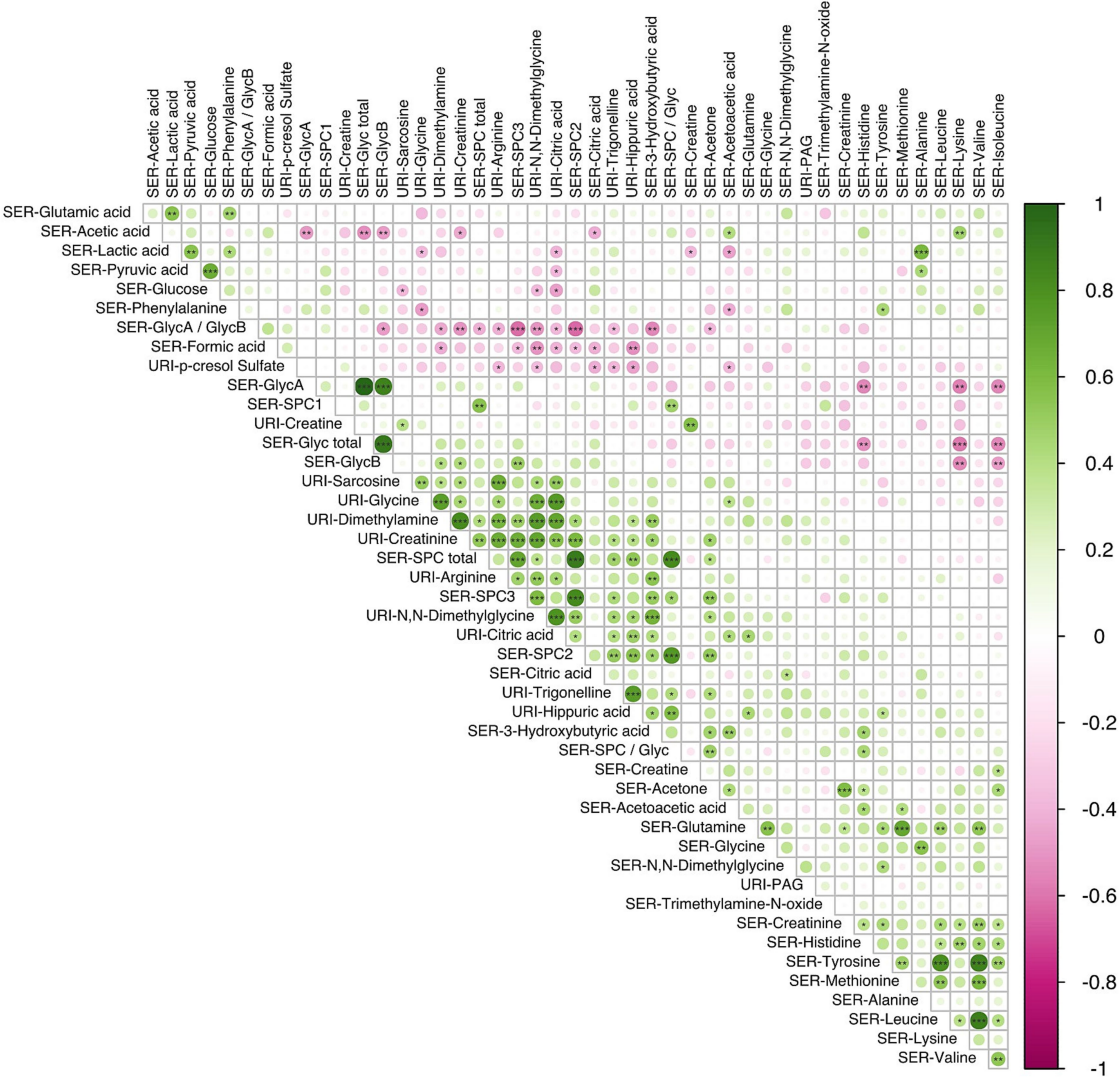
Correlation analysis using the Spearman pairwise rank method showing relationship between small molecules identified in serum (*n* = 34) and urine (*n* = 12) samples obtained during the third trimester of pregnancy (16 LMDA and 15 HMDA). The molecules are clustered using the angular order of the eigenvectors. Associations ranked from 1 (green), representing positive correlation, to −1 (pink), representing negative correlation (significance level according to Spearman correlation coefficients: *p* < 0.001 = ***; *p* < 0.01 < **; *p* < 0.05 = *). SER, serum; URI, urine; SPC, supramolecular phospholipid composite; Glyc, acute phase glycoprotein glycan; PAG, phenylacetylglutamine.

Correlations between individual metabolites are highlighted in Figure 5 (see Supplementary Figure S7 for metabolite correlation matrices stratified according to diet). A number of direct correlations were observed between metabolites that were related by compound class or pathway. For example, GlycA with GlycB for both dietary patterns (*p* < 0.001); SPC2 with SPC3 (*p* < 0.001); serum branched chain amino acids (BCAAs) (valine, leucine, isoleucine) with tyrosine and serum glucose with pyruvate (*p* < 0.001). In the LMDA group, a strongly significant anti-correlation was found between lysine (serum) and the two GlycA and GlycB (serum) (*p* < 0.05).

The three calculated SPC components, although part of the same composite phospholipid peak, showed a different correlation structure with other serum and urinary metabolites. SPC1 correlated with the total SPC concentration but showed little or no correlation with SPC 2 and SPC3, which were strongly correlated with each other (*p* < 0.01) (Figure 5). This is consistent with the lipoproteins that contribute to the SPC components, with SPC1 being composed mainly of HDL4, SPC2 mainly of HDL1-3, and SPC3 of LDL particles. In keeping with this observation, the total SPC concentration is inversely correlated with the total Glyc concentration, but positively correlated with metabolites associated with plant-based diets, such as hippurate. Other notable correlations were observed between other various urinary gut metabolites, hippuate inversely associated with paracresol sulfate and formate, and positively correlated with PAG and dimethylamine.

**Figure 6:**
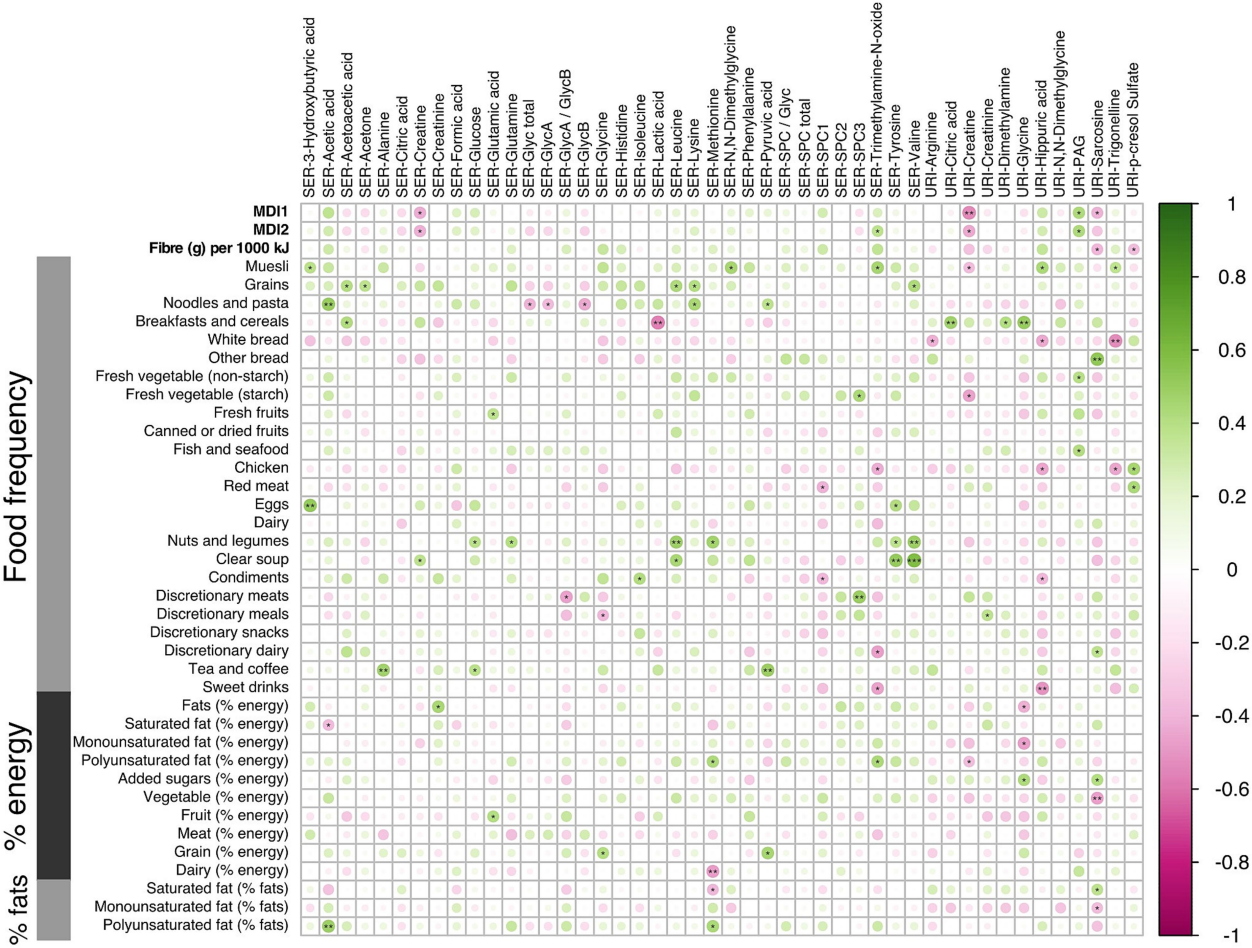
Correlation analysis using the Spearman pairwise rank method for dietary scores and small molecules during the third trimester of pregnancy (16 LMDA and 15 HMDA). Associations ranked from 1 (green), representing positive correlation, to −1 (pink), representing negative correlation (significance level according to Spearman correlation coefficients: *p* < 0.001 = ***; *p* < 0.01 < **; *p* < 0.05 = *). SER, serum; URI, urine; SPC, supramolecular phospholipid composite; Glyc, acute phase glycoprotein glycan; PAG, phenylacetylglutamine.

These correlations between microbial metabolites were generally stronger for the LMDA group than the HMDA group (see Supplementary Figures S7A,B). A correlation plot (Figure 6) was created to allow for associations to be drawn between specific metabolites and particular dietary components identified in the FFQ’s (see also Supplementary Figures S6, S7). Of particular interest were the food correlations with SPC1, corresponding predominantly to small HDL phospholipid particles (HDL-4), which is recognised as a protective lipoprotein entity in cardiovascular disease (49). SPC1 was mildly, although not significantly, negatively associated with saturated fat intake, and significantly negatively associated with red meat (*p* < 0.05) and condiments (*p* < 0.05). SPC1 was non-significantly positively associated with vegetable intake (% energy). Conversely, SPC3, reflecting LDL phospholipid particles, was positively correlated with discretionary meats (*p* < 0.01), and starch vegetables, including potato (*p* < 0.05).

Of note, the serum amino acids valine, tyrosine, glutamine, methionine and leucine were associated with increased fat intake and with nuts and legumes. Inflammatory markers GlycA and GlycB demonstrated negative associations with cottage cheese, noodles and pasta. Urinary hippurate, a gut microbial-host co-metabolite, was strongly associated with some vegetables, fruit and muesli, and had strong negative associations with white bread, chicken and sweet drinks, as well as condiments. Excretion of acetate, a short chain fatty acid, predominantly of microbial origin, was strongly associated with dietary intake of polyunsaturated fats, noodles and some fruits, while being negatively correlated with saturated fats.

## 4 Discussion

This study compared the metabolic phenotypes of women whose dietary patterns were self selectively aligned, or not, with the Mediterranean diet (characterised by consumption of fruits, vegetables, nuts, fish, olive oil and wholegrains, with minimal intake of processed foods, red meat and saturated fats). The diet-related differences between the LMDA and HMDA groups were reflected in both the urine and the serum profiles, indicating that alignment with the Mediterranean diet is associated with a specific metabolic phenotype during pregnancy. The MDI was inversely associated with inflammatory markers GlycA and GlycB but positively associated with SPC1 (reflecting HDL-4 concentration), which suggests that the women following the Mediterranean diet had a lower level of systemic inflammation.

Comparison of the FFQ data, between trimesters 2 and 3 showed a remarkable consistency in terms of reporting of dietary composition with clear differences in the food and nutrient composition between the two dietary groups relating to high and low alignment to the Mediterranean diet at both time points. Although the MDI scores were used to select participants who had no more than 2 points difference, the FFQ is made up of 40 different food groups and so the similarity between the two time points in terms of the finer dietary profile was noteworthy. The MDI showed direct correlation across both time points with the urinary gut microbial metabolites hippurate, phenylacetylglutamine and acetate and inverse correlations with both serum and urine creatine and with sarcosine, which are associated with more meat-based diets.

### 4.1 An inflammatory metabolic profile is associated with low alignment with a Mediterranean dietary pattern

One of the most striking differences between the low and high alignment with Mediterranean diet in the current study was the presence of higher concentrations of the inflammatory markers GlycA and GlycB in the serum of women in the LMDA group, as well as the difference in SPC patterns between the groups. The ^1^H NMR signals from GlycA and GlycB underlie a combination of acute phase glycoproteins, with alpha-1-acid glycoprotein being the most prominent but also encompassing haptoglobin, transferrin and alpha-1-antitrypsin (25). GlycA and GlycB are known to be highly correlated markers of inflammation and have been shown to be associated with a wide range of inflammatory health conditions (50). GlycA has been reported to be more sensitive and more stable than the more commonly used high sensitivity C-reactive protein, used as a gold standard marker of inflammation (34), and is elevated in obesity (24). Both GlycA and GlycB are associated with coronary heart disease (51) and other vascular inflammatory states (50). One study demonstrated that GlycA is associated with all-cause mortality (52, 53).

The supramolecular phosphocholine composite (SPC) signal, which contains the choline headgroups of lysophosphatidylcholines on glycoproteins and phospholipids in LDL and HDL, has been shown to be negatively correlated with inflammatory conditions (41, 43). Clear differences in the SPC patterns between the dietary adherence groups indicated that the SPC1, the resonance deriving from cardioprotective HDL-4 (54), was increased for those in the HMDA group and was negatively correlated with many dietary components that are not present in the Mediterranean dietary pattern, such as intake of red meat, discretionary foods (chocolate, chips etc.) and sweet drinks (such as soft drinks and juices). Conversely, SPC2 and SPC3 were increased in those participants in the LMDA group and this was associated with increasing discretionary meats (ham, bacon, salami etc.). The SPC3 signal derives from LDL phospholipids, which are generally considered to be proinflammatory, while SPC2 derives from larger HDL(1-3) phospholipid particles. This study demonstrates for the first time that SPC signals are associated with specific dietary patterns and may be manipulated by a change in eating habits.

These findings of metabolite profiles consistent with lower inflammation in the HMDA group are concordant with previous studies demonstrating that plant-based diets consumed during pregnancy by overweight or obese mothers have been associated with a reduction in the low-grade inflammation and with a higher gut microbial diversity. This is likely related to the increased fibre content in the plant-based diet (55). Since GlycA and GlycB are positively associated with LMDA but were not strongly associated with any specific dietary parameter, this indicates that the Mediterranean diet pattern (whole grains, pasta and noodles, fresh fruit, and vegetables) suggests that the whole dietary profile high in fibre is more important than individual components. Several weak associations between specific nutrients/foods and the acetylated glycoproteins indicated that the balance of animal protein to carbohydrate content may be important and is worth pursuing further. These results suggest that a Mediterranean diet may support both mother and child physically via a reduction in inflammation.

### 4.2 Adherence to a Mediterranean diet pattern affects the gut microbial metabolite phenotype

Hippurate was the strongest metabolic differentiator between the urine profiles of the HMDA and LMDA groups. Produced as a co-metabolite of microbial (production of benzoic acids) and human (glycine conjugation in the hepatic mitochondria) metabolism, hippurate is associated with reductions in blood pressure, obesity, visceral fat, and non-alcoholic fatty liver disease in adult populations (49, 56). Hippurate is an established surrogate marker for metabolic health, gut function and microbial diversity (57, 58). The composition of the gut microbiome is important during pregnancy as it has demonstrated impacts on foetal outcomes, as well as long-term health implications for both mother and child including the regulation of glucose metabolism and gestational diabetes, obesity, and risk of preeclampsia during pregnancy (59). Our study demonstrated significantly increased urinary hippurate levels in those closely adhering to the Mediterranean diet. Hippurate intake is known to be associated with higher intakes of whole grains, coffee, fruit and vegetables, and a diet which is overall high in fibre and polyphenols (56, 57, 60, 61). As such, higher levels of hippurate are generally associated with a healthier eating pattern and leaner body mass (56). Hippurate has previously been associated with dietary fibre intake (61), again concordant with the dietary intake of those on a Mediterranean Diet. However, it should be mentioned that not all healthy dietary patterns are associated with higher levels of hippurate. For example, although the traditional Japanese diet is known to be inversely associated with cardiovascular disease and is high in plant-based phenolics, hippurate excretion, in general, is lower in Japanese individuals compared to participants from the U.K., U.S.A and People’s Republic of China (61).

Hippurate directly correlated with phenylacetylglutamine, a product of aromatic amino acid breakdown by the colonic bacteria. Phenylacetylglutamine correlated with the MDI and was specifically associated with fresh vegetables, fruits and fish, adding weight to the observed impact of the Mediterranean diet on the gut microbiome. Increased levels of phenylacetylglutamine have previously been reported in individuals showing high adherence to the Mediterranean diet (62) and after consumption of foods that are rich in polyphenols (63).

We observed further dietary impact on microbial metabolites with higher concentrations of acetate, a short chain fatty acid typically produced from the metabolism of indigestible carbohydrates, or fibre, found in the serum of women with high adherence to a Mediterranean diet pattern. The short chain fatty acids are associated with gut microbial function and this relationship between the Mediterranean diet and circulating acetate levels has been reported previously (64). High fibre foods include fruits and vegetables, wholegrain foods, nuts and seeds, all of which are highly prevalent in the Mediterranean diet. Circulating acetate concentrations are associated with lower body weight, reduced risk of cardiovascular disease and increased immune functioning (65, 66). In addition, acetate levels during pregnancy may have a lasting impact on the developing foetus. Hu and colleagues found that low serum acetate was associated with increased risk of pre-eclampsia (67). Similarly, Brantsæter and colleagues found a reduced risk of pre-eclampsia in pregnant women who regularly consumed probiotic products, which were capable of increasing acetate levels in the gut environment (68). The relationship between fibre intake and acetate levels during pregnancy and the subsequent impact on offspring health, specifically ectopic conditions have been further demonstrated in mouse models (69). In the current study we found a modest association, although not significant, between serum acetate and percentage of dietary polyunsaturated fats, whereas there was an inverse correlation between serum acetate and saturated fats (*p* < 0.01). This supports the concept that high dietary fibre intake is likely indicative of a healthy dietary profile.

Adding to the body of evidence that the Mediterranean diet beneficially influences the gut microbiome is the observed inverse correlation between serum GlycA and serum acetate, which were present in higher and lower relative concentrations, respectively, in the samples of women in the LMDA group. GlycA has been reported to have an inverse correlation with dietary fibre and gut microbiome diversity (20).

### 4.3 Creatine and meat intake is associated with low adherence to a Mediterranean diet pattern: balance of animal to plant-based diets

Creatine was noted as the most significant serum metabolite driving the difference between the two diet groups, being present in higher concentrations in the serum profiles of the LMDA group and a strongly correlation between serum and urinary creatine was identified. Creatine can be derived from dietary sources, such as fish, meat and dairy, or can be synthesized endogenously (70). Creatine is required to facilitate the developing foetus, as well as the uterine and placental tissues, particularly in the third trimester, and is necessary for adequate foetal growth (71). Human studies demonstrate that pregnant women have lower serum levels of creatine by approximately 35%, compared to non-pregnant women, and the excretion of creatine reduces as the pregnancy progresses. Although, unlike other studies (70) we find no significant correlation between serum creatinine and red meat in the current study, serum creatine was weakly associated with all types of meat and fish in the LMDA group but not the HMDA group (Supplementary Figure S3) and urine creatinine was inversely correlated with vegetable and fruit intake in the LMDA group. Consumption of meat is associated with increased excretion of urinary creatine and creatinine (70) but the effects are relatively small with a 13% increase in creatinine excretion observed following a meal of cooked red meat (72). We found a small but significant increase in serum concentrations of creatine in the HMDA and trend towards higher urinary creatine concentrations in the LMDA group. The failure to reach significance is likely due to the relatively small number of participants enrolled in the study. The correlation of urinary creatine to urinary creatinine, sarcosine, dimethylglycine and dimethylamine and serum creatine adds confidence in the association of these metabolites with protein intake. Considering the combined urine and serum metabolomes, we observe different patterns between the HMDA and LMDA reflective of discordance in the ratio of meat to plant-based dietary protein. For example, serum creatine was inversely associated with trigonelline, which is an indicator of bean and pea consumption (73, 74). A low creatine to trigonelline ratio is therefore consistent with adherence to a Mediterranean dietary pattern, as there is a focus on plant-based protein sources such as legumes. Trigonelline has been associated with a range of health benefits, and is credited with hypoglycaemic, hypolipidemic, and neuroprotective properties (75, 76). As such, a Mediterranean dietary pattern, which is lower in meat consumption than a Western dietary pattern, is likely to rely on a greater percentage of plant-based sources of protein.

### 4.4 Association of fish intake, urinary methylamines, and the Mediterranean diet

Consistent with a Mediterranean dietary pattern, participants reported higher consumption of fish in the HMDA group. Urinary trimethylamine-*N*-oxide (TMAO) and its precursor trimethylamine are markers of fish consumption, particularly deep dwelling marine fish (77) but are also synthesized from bacterial degradation of dietary choline and carnitine found in red meats (56, 77). Less commonly, urinary TMAO can also be related to legume intake (78). Urinary and serum TMAO have been proposed as biomarkers of cardiovascular disease (79), however caution must be exercised in interpreting its role in dietary health. This is due to the fact that there are dietary sources of this metabolite, including fish (77), which is generally associated with alignment to a Mediterranean-style diet. In the current study, TMAO was positively associated with increased polyunsaturated fats, muesli intake, and nuts and legumes. This indicates that the source of choline in the HMDA group is more likely to be from plant-based sources, such as legumes and whole grains, and that fish intake, although increased in the HMDA group, does not significantly account for urinary or serum concentrations of methylamines in this case. In the current study the median spectrum of urinary TMAO (Supplementary Figure S6E) was higher in the HDMA group, but this difference was not significant in either the univariate or multivariate models due to the relatively small group size and the fact that TMAO was overlapped with multiple signals from betaine and choline species. In contrast to our observation, De Filippis and co-workers found that adherence to a Mediterranean diet was linked to lower urinary trimethylamine-*N*-oxide concentrations (80).

### 4.5 The Mediterranean diet is associated with differences in amino acid metabolism

Alignment with a Mediterranean diet was associated with generally lower levels of serum amino acids, with the exception of glycine, which associated with the HMDA group. The Branched Chain Amino Acids (BCAA’s) valine and leucine were found in higher concentrations in the HMDA group, as was tyrosine. These metabolites showed significant inter-correlation and were also associated with intake of legumes and nuts, key components of the Mediterranean diet. The implications of BCAA’s in chronic disease are, as yet, unclear, with contradictory studies indicating for their benefit or detriment. However, the consensus from most studies is that BCAA’s play an anti-inflammatory role in the body and can reduce levels of proinflammatory mediators interleukin-6 and cyclooxygenase-2 (81). Indeed, one study which examined the metabolome, as omnivorous participants moved to a vegan diet for 48 h, demonstrated that BCAA’s were notably increased in the vegan metabolome, as part of an overall improved metabolic profile (82).

Other differences between the urine profiles of the two diet groups were the lower concentrations of alanine in the LMDA group along with two products of threonine metabolism, 4-deoxyerythronic acid and 4-deoxythreonic acid. Urinary concentrations of these products of threonine catabolism have been shown to increase over the course of pregnancy (83) and have been found to be correlated with BMI (84). In non-pregnant cohorts 4-deoxythreonic acid is associated with major depressive disorder (85). In a dietary study comparing the impact of a Mediterranean diet with that of an ultra-processed food diet, 4-deoxythreonic acid was inversely associated with the Mediterranean diet (85), consistent with the results we observe here.

### 4.6 Limitations

The main limitation of this study was the relatively small sample size of the cohort when stratified by diet, which prohibits overinterpretation of the results. Not all participants completed the food frequency questionnaire at all time points and as such the Mediterranean diet screening that was conducted may not be fully reliable. The cohort were self-selected and while every effort was made to ensure there was no bias between the two diet groups, because the groups were self-selected and the diets were not randomised, it is possible that there is an inherent bias between the two groups. However, there was no statistical difference after adjusting for multiple testing in age, parity, education, work status, pre-pregnancy weight/BMI, and socioeconomic indexes for areas factors. On the other hand, because the diets were self-selected, it could be possible that adherence may be better than when randomising participants to different diets. Nevertheless, using the FFQ’s to confirm the expected food correlations between the two groups indicated consistency of the dietary patterns established by the MDQ within each group and between timepoints. Since it would have been difficult to obtain ethics for fasting samples in this pregnancy cohort, the biological samples were not taken in a fasting state, which may distort the biofluid composition based on the last meal consumed. However, the differences in metabolic phenotype between the two groups was convincing and based on the metabolite-metabolite and metabolite-diet correlations, the metabolic consequences were in keeping with a lower inflammatory profile in the HMDA group.

## 5 Conclusion

We demonstrated a clear difference between the metabolite profiles of pregnant women who had a high versus low Mediterranean diet score. These metabolite profiles align with their reported choice of food. High adherence to a Mediterranean diet was associated with lower serum markers of inflammation, creatine and an altered amino acid profile, together with increased concentrations of gut-microbial metabolites, indicating greater functional diversity associated with adherence to a Mediterranean diet. This may have long term health benefits for both mother and child. The combination of serum inflammatory markers and hippurate as a marker of dietary fibre intake may provide an effective biomarker panel for assessing adherence to Mediterranean diet in pregnancy.

## Data availability statement

The original contributions presented in the study are included in the article/Supplementary material, further inquiries can be directed to the corresponding authors.

## Ethics statement

The studies involving humans were approved by ORIGINS Scientific Committee and Project Management Group (Application ID: ND01905); Ramsay Health Care Human Research Ethics Committee (Protocol number: ND01905). The studies were conducted in accordance with the local legislation and institutional requirements. The participants provided their written informed consent to participate in this study.

## Author contributions

ND’V: conceptualization and methodology. CR, SL, SE, and JW: investigation. CR, GF, and EH: writing – original draft preparation. EH, GF, JN, ND’V, CC, and TO’S: validation, resources, and writing – review and editing. ND’V and JN: funding acquisition. ND’V, EH, and GF: supervision. All authors contributed to the article and approved the submitted version.

## Footnotes

^1^github.com/tkimhofer/metabom8
