# Supplementary material for: Altered dietary behaviour during pregnancy impacts systemic metabolic phenotypes
Source: Front Nutr. 2023 Dec 4;10:1230480. doi: 10.3389/fnut.2023.1230480 (PMC10725961; doi:10.3389/fnut.2023.1230480)
Supplement: Supplementary file 1 [file Data_Sheet_1.docx]

***Supplementary Material***

**1 Supplementary Methods**

##
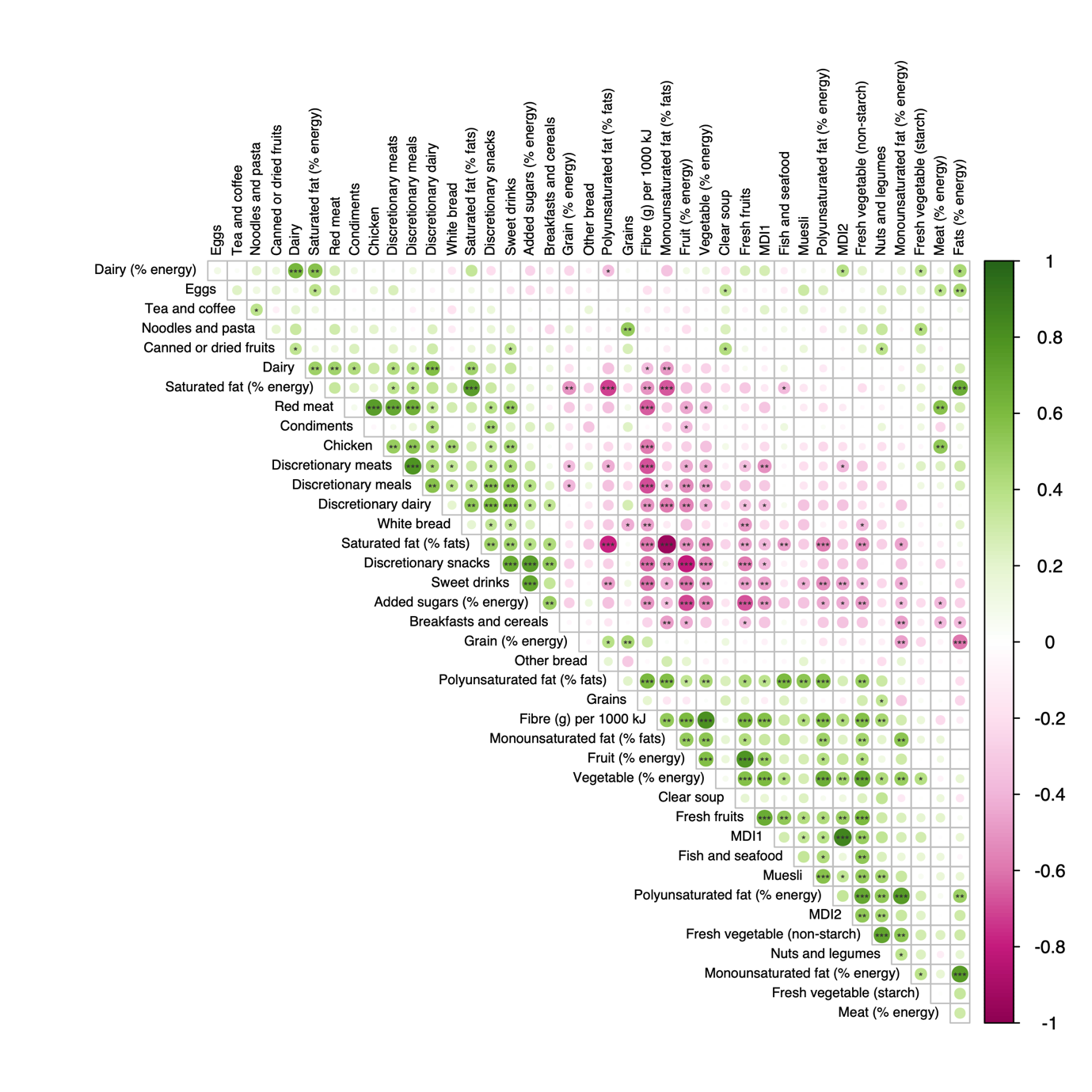


**Supplementary Figure 1**. Correlation analysis using spearman pairwise rank method showing relationship between dietary components. A set of 40 variables were analysed including the MDI score obtained at timepoint 1 and 2, the amount of fibre expressed as grams per 1000 kJ, 24 food frequency variables, 10 variables related to percentage of energy, and three variables related to percentage of fat intake. Significance level assessed using spearman correlation coefficient displaying significant levels (P < 0.001 = ***; P < 0.01 < **; P < 0.05 = *), associated small molecules clustered using the angular order of the eigenvectors. Associations ranked from 1 (green) representing positive correlation to -1 (pink) representing negative correlation.

**(A)**

**(B)**

**Supplementary Figure 2** Correlation analysis using spearman pairwise rank method for samples obtained during the third trimester of pregnancy. Showing relationship between metabolites in serum (n = 34) and urine (n = 12) and dietary scores. **(A)** Participants with LMDA (n = 16) and **(B)** participants with HMDA (n = 15). Significance level assessed using spearman correlation coefficient displaying significant levels (P < 0.001 = ***; P < 0.01 < **; P < 0.05 = *). Associations ranked from 1 (green) representing positive correlation to -1 (pink) representing negative correlation.

** Supplementary Figure 3**. Correlation analysis using spearman pairwise rank method showing relationship between dietary components and metabolites in serum (n = 34) and urine (n = 12) during the third trimester (16 LMDA and 15 HMDA). A set of 85 diet variables were analysed including the MDI score obtained at timepoint 1 and 2, the amount of fibre expressed as grams per 1000 kJ, 59 food frequency variables, 10 variables related to nutrient intake, 10 variables related to percentage of energy, and three variables related to percentage of fat intake. Significance level assessed using spearman correlation coefficient displaying significant levels (P < 0.001 = ***; P < 0.01 < **; P < 0.05 = *). Associations ranked from 1 (green) representing positive correlation to -1 (pink) representing negative correlation.

**Supplementary Figure 4**. Univariate analysis of nine selected urine metabolites obtained by Bruker IVDr for samples collected at 36 weeks. LMDA (n = 22) shown in red and HMDA (n = 23) shown in blue.


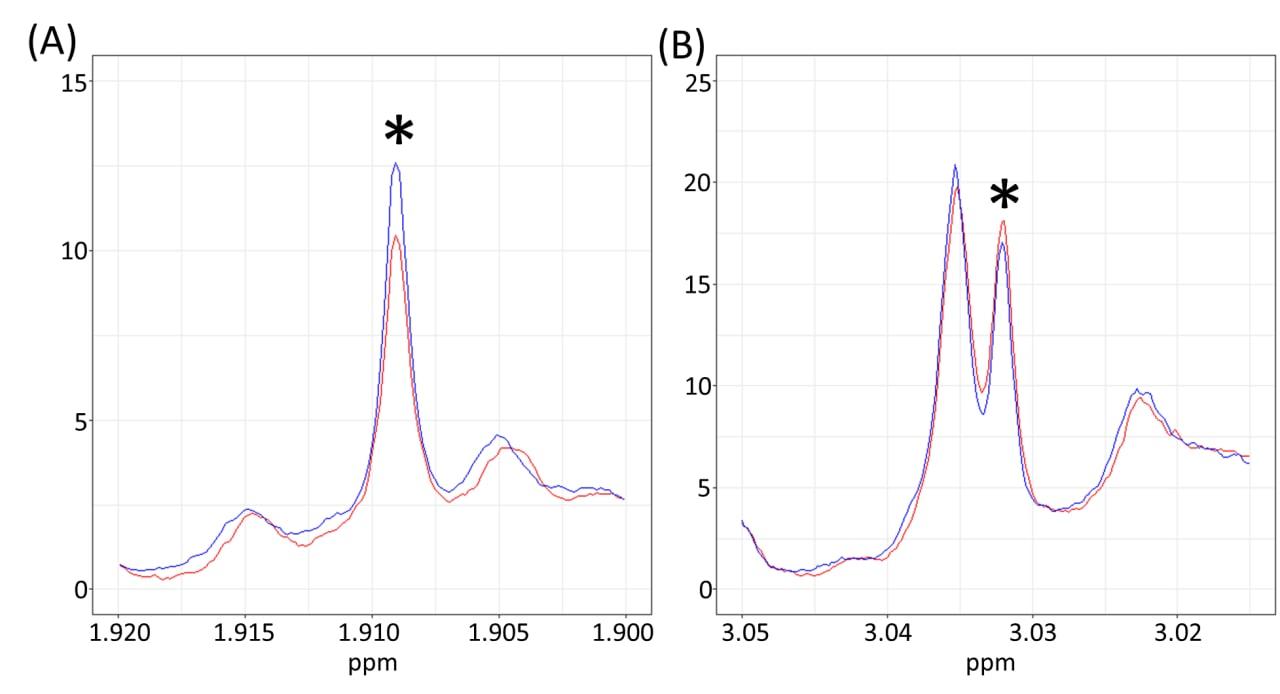


**Supplementary Figure 5**. Median *^1^H CPMG NMR* spectra for serum samples obtained at 36 weeks (LMDA shown in red and HMDA shown in blue). Median *1D ^1^H NOESY NMR* for serum. HMDA in blue and LMDA in red. **(A)** Acetic acid and **(B)** Creatine.


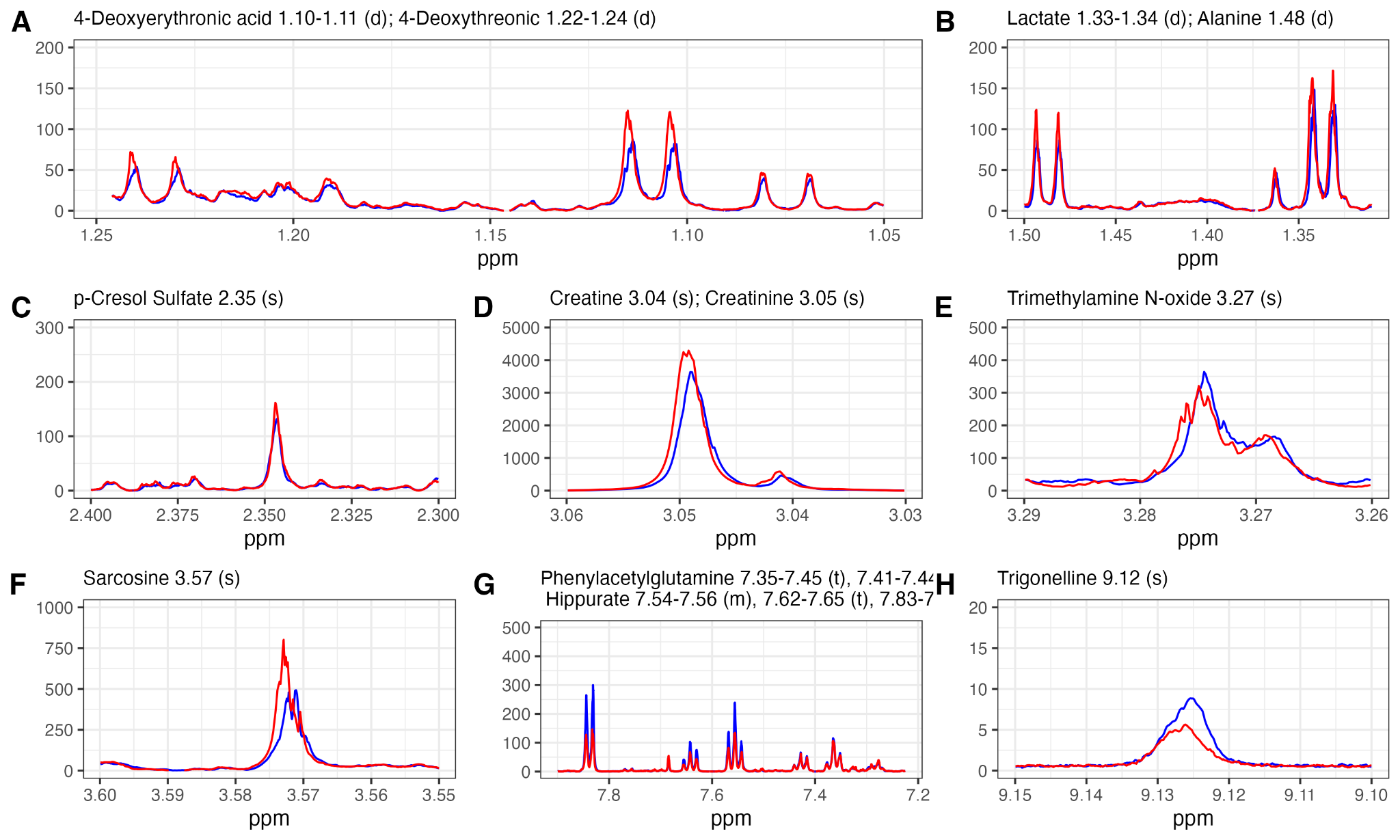


**Supplementary Figure 6**. Median standard one-dimensional (*1D) ^1^H NMR* spectra for urine samples obtained at 36 weeks. LMDA (n = 22) shown in red and HMDA (n = 23) shown in blue.

**(A)**

**(B)**

**Supplementary Figure 7**. Correlation analysis using spearman pairwise rank method showing relationship between metabolites in serum (n = 34) and urine (n = 12) for samples obtained at 36 weeks. **(A)** Participants with LMDA (n = 16) and **(B)** participants with HMDA (n = 15). Significance level assessed using spearman correlation coefficient displaying significant levels (P < 0.001 = ***; P < 0.01 < **; P < 0.05 = *), associated small molecules clustered using the angular order of the eigenvectors. Associations ranked from 1 (green) representing positive correlation to -1 (pink) representing negative correlation.

**3**  **Supplementary Tables**

*Supplementary Table 1: The 13-point mediterranean diet adherence screener*

| **Food consumption frequency/Food intake habits** | **1 point** |
| --- | --- |
| Do you use olive oil as the principal source of fat for cooking? | Yes |
| How many *tablespoons of olive oil do you consume per day (including that used in frying, salads, meals eaten away from home, etc.)? | ≥ 4 Tbsp |
| How many servings of vegetables do you consume per day? Count garnish and side servings as 1/2 point; 3. a full serving is 200 g. | ≥ 2 |
| How many pieces of fruit (including fresh-squeezed juice) do you consume per day? ≥ 3 | ≥ 3 |
| How many servings of red meat, hamburger, or sausages do you consume per day? A full serving is 100-150 g. | < 1 |
| How many servings (12 g) of butter, margarine, or cream do you consume per day? | < 1 |
| How many carbonated and/or sugar-sweetened beverages do you consume per day? | < 1 |
| How many servings (150 g) of pulses do you consume per week? | ≥ 3 |
| How many servings of fish/seafood do you consume per week? (100-150 g of fish, 4-5 pieces or 200 g of seafood) | ≥ 3 |
| How many times do you consume commercial (not homemade) pastry such as cookies or cake per week? | < 2 |
| How many times do you consume nuts per week? (1 serving = 30 g) | ≥ 3 |
| Do you prefer to eat chicken, turkey or rabbit instead of beef, pork, hamburgers, or sausages? | Yes |
| How many times per week do you consume boiled vegetables, pasta, rice, or other dishes with a sauce of 13. tomato, garlic, onion, or leeks sauted in olive oil? | ≥ 2 |

*Supplementary Table 2: Grouped Dietary Components from Food Frequency Questionnaire*

| **Group Name** | **Dietary Items** |
| --- | --- |
| Sweet Drinks | Daily soft drinks, diet soft drinks, soft drink, juice, cordials, flavoured milk. |
| Discretionary snacks | Hash browns, potato scallops, twisties, corn chips, burger rings, potato crisps, water iceblock, chocolate, lollies, jelly, home fries, muffins (& etc.), cakes (& etc.), sweet pastries, puddings, sweet biscuits, cream biscuits, savoury biscuits, snack noodles, fruit bars, snack bars, muesli bars. |
| White bread | White bread. |
| Discretionary meals | Takeaway meals, takeaway fries, pizza. |
| Chicken | Chicken (with and without vegetables), plain chicken, crumbed chicken. |
| Condiments | low fat dressing, mayonnaise, nuts, jam, honey, golden syrup (& etc.), peanut butter, Nutella, Vegemite, Promite, Marmite, BBQ sauce, tomato sauce. |
| Discretionary meats | Pies, sausage rolls, chiko roll, hot dogs, bacon, ham, devon, salami, sausages, hamburgers, frankfurts. |
| Red meat | Mince, lamb (with and without vegetables), plain meat (with and without vegetables), pork (with and without vegetables), plain pork (with and without vegetables), liver. |
| Dairy | Milk, plain milk, yoghurt, cottage cheese, cheese. |
| Discretionary dairy | Creamy ice block (e.g. Magnum, Cornetto), creamy soup, frozen yoghurt, icecream, cream. |
| Canned or dried fruits | Dried fruit, canned fruit. |
| Tea and coffee | Tea, coffee. |
| Noodles and pasta | Spaghetti, lasagne, pasta bake, yellow egg noodles, rice noodles. |
| Eggs | Boiled and scrambled. |
| Fresh vegetables (starch) | Potato, sweet potato. |
| Nuts and legumes | Peas, soy beans, tofu, baked beans, lentils, chickpeas, split peas, green beans. |
| Fresh vegetables (non-starch) | Cauliflower, spinach, cabbage (& etc.), broccoli, carrots, zucchini (& etc), capsicum, corn (& etc.), mushrooms, tomatoes, lettuce, celery (& etc.), avocado, onion (& etc.). |
| Muesli | Muesli. |
| Breakfasts and cereals |  |
| Fish and seafood | Crumbed fish, fresh fish, canned fish, other seafood. |
| Fresh fruits | Fruit, fruit salad, apple (& etc.), orange (& etc.), banana, peach (& etc.), mango (& etc.), pineapple, grape, melon. |
| Clear soup | Clear soup with rice or noodles. |
| Grains | Porridge, rice, couscous, burghul, wholegrain bread. |
| Other bread | Other bread. |

*Supplementary Table 3: Dietary Variables Collected*

| **Group Name** | **Variables** |
| --- | --- |
| MDI scores | Collected at weeks 20, 28, 36 |
| 24 FFQ variables | See supplementary table 1 |
| Percentage of energy from: | Protein; carbohydrates; fats, saturated fats; polyunsaturated fats; monounsaturated fats; added sugars; vegetables; fruit; meat |
| Percentage of fat intake | Saturated fat; polyunsaturated fat, monounsaturated fat |

*Supplementary Table 4A: p-values for dietary components in Trimester 2*

| Dietary component | OPLS loadings | Cliff’s delta | p-value | Adjusted p-value |
| --- | --- | --- | --- | --- |
| Orange_etc | 0.174 | 0.90 | 1.93x10^-4^ | 1.60x10^-2^ |
| Fruit | 0.225 | 0.86 | 4.09x10^-4^ | 1.70x10^-2^ |
| peaddedsugars | 0.189 | -0.82 | 1.09x10^-3^ | 2.03x10^-2^ |
| Sweet.drinks | 0.207 | -0.81 | 1.22x10^-3^ | 2.03x10^-2^ |
| Fresh_fish | 0.206 | 0.82 | 1.93x10^-3^ | 2.03x10^-2^ |
| Tomatoes | 0.166 | 0.75 | 2.00x10^-3^ | 2.77x10^-2^ |
| White.bread | 0.150 | -0.60 | 3.68x10^-3^ | 4.37x10^-2^ |
| Disc.snacks | 0.182 | -0.69 | 6.09x10^-3^ | 6.32x10^-2^ |
| Fibre | 0.124 | 0.65 | 9.80x10^-3^ | 7.55x10^-2^ |
| DHA | 0.131 | 0.65 | 9.80x10^-3^ | 7.55x10^-2^ |
| Pefruit | 0.180 | 0.64 | 1.03x10^-2^ | 7.55x10^-2^ |
| Avocado | 0.154 | 0.63 | 1.09x10^-2^ | 7.55x10^-2^ |
| Grains | 0.134 | 0.62 | 1.39x10^-2^ | 8.49x10^-2^ |
| Other_seafood | 0.143 | 0.57 | 1.45x10^-2^ | 8.49x10^-2^ |
| Zucchini _etc | 0.128 | 0.60 | 1.57x10^-2^ | 8.49x10^-2^ |
| Carrots | 0.125 | 0.53 | 1.64x10^-2^ | 8.49x10^-2^ |
| Apple_etc | 0.146 | 0.55 | 2.07x10^-2^ | 9.57x10^-2^ |
| Celery_etc | 0.151 | 0.57 | 2.09x10^-2^ | 9.57x10^-2^ |
| Noodles | 0.108 | 0.56 | 2.25x10^-2^ | 9.57x10^-2^ |
| Yoghurt | 0.130 | 0.56 | 2.31x10^-2^ | 9.57x10^-2^ |
| Nuts…legumes | 0.128 | 0.56 | 2.69x10^-2^ | 0.106 |
| Canned_fruit | 0.133 | -0.40 | 2.90x10^-2^ | 0.109 |
| Spinach | 0.149 | 0.53 | 3.04x10^-2^ | 0.110 |
| Red.meat | 0.131 | -0.53 | 3.43x10^-2^ | 0.115 |
| Muesli | 0.134 | 0.51 | 3.46x10^-2^ | 0.115 |
| Disc.Meals | 0.126 | -0.52 | 3.96x10^-2^ | 0.126 |
| Crumbed_chicken | 0.140 | -0.49 | 4.10x10^-2^ | 0.126 |
| Disc.Dairy | 0.134 | -0.51 | 4.30x10^-2^ | 0.128 |
| PePFA | 0.109 | 0.48 | 4.49x10^-2^ | 0.128 |
| Peveg | 0.140 | 0.50 | 4.62x10^-2^ | 0.128 |
| Pedairy | 0.120 | 0.49 | 5.00x10^-2^ | 0.134 |

*Supplementary Table 4B: p-values for dietary components in Trimester 3*

| Dietary component | OPLS loadings | Cliff’s delta | p-value | Adjusted p-value |
| --- | --- | --- | --- | --- |
| Peveg | 0.212 | 0.69 | 1.00x10^-3^ | 5.17x10^-2^ |
| Sweet.drinks | 0.209 | -0.68 | 1.25x10^-3^ | 5.17x10^-2^ |
| Peaddedsugars | 0.158 | -0.57 | 6.48x10^-3^ | 5.33x10^-2^ |
| Fruit | 0.157 | 0.56 | 6.04x10^-3^ | 5.33x10^-2^ |
| Disc.Dairy | 0.176 | -0.58 | 5.46x10^-3^ | 5.33x10^-2^ |
| Milk | 0.190 | -0.55 | 4.85x10^-3^ | 5.33x10^-2^ |
| Crumbed_chicken | 0.169 | -0.55 | 7.06x10^-3^ | 5.33x10^-2^ |
| Disc.Meat | 0.177 | -0.57 | 6.61x10^-3^ | 5.33x10^-2^ |
| Spinach | 0.123 | 0.60 | 3.06x10^-3^ | 5.33x10^-2^ |
| Cabbage_etc | 0.105 | 0.58 | 5.27x10^-3^ | 5.33x10^-2^ |
| Celery_etc | 0.195 | 0.63 | 2.18x10^-3^ | 5.33x10^-2^ |
| Zucchini_etc | 0.089 | 0.54 | 8.04x10^-3^ | 5.56x10^-2^ |
| Pefruit | 0.181 | 0.51 | 1.47x10^-2^ | 8.43x10^-2^ |
| Carrots | 0.158 | 0.49 | 1.46x10^-2^ | 8.43x10^-2^ |
| Apple_etc | 0.110 | 0.50 | 1.52x10^-2^ | 8.43x10^-2^ |
| pePFA | 0.135 | 0.46 | 1.75x10^-2^ | 8.56x10^-2^ |
| pfSFA | 0.146 | -0.50 | 1.73x10^-2^ | 8.56x10^-2^ |
| Pfpolyfats | 0.121 | 0.48 | 2.11x10^-2^ | 9.21x10^-2^ |
| Nuts…legumes | 0.104 | 0.49 | 2.07x10^-2^ | 9.21x10^-2^ |
| TFA | 0.156 | -0.48 | 2.43x10^-2^ | 9.31x10^-2^ |
| Muesli | 0.079 | 0.44 | 2.48x10^-2^ | 9.31x10^-2^ |
| Breakfast_cereal | 0.115 | -0.46 | 2.58x10^-2^ | 9.31x10^-2^ |
| Avocado | 0.142 | 0.47 | 2.31x10^-2^ | 9.31x10^-2^ |
| Tomatoes | 0.128 | 0.44 | 3.08x10^-2^ | 0.107 |
| Disc.Snacks | 0.186 | -0.43 | 3.97x10^-2^ | 0.117 |
| Pumpkin | 0.086 | 0.42 | 4.01x10^-2^ | 0.117 |
| Sweet_potato | 0.092 | 0.41 | 4.08x10^-2^ | 0.117 |
| Cauliflower | 0.095 | 0.40 | 4.06x10^-2^ | 0.117 |
| Melon | 0.109 | 0.42 | 3.97x10^-2^ | 0.117 |
| Pineapple | 0.119 | 0.42 | 4.23x10^-2^ | 0.117 |
| Fresh_fish | 0.087 | 0.41 | 4.40x10^-2^ | 0.118 |
| pFMA | 0.136 | 0.42 | 4.71x10^-2^ | 0.122 |

*Supplementary Table 5A: Foods Associated with HMDA*

| Food/nutrient | pval trimester 2 | pval trimester 3 | Cliff's delta T2 | Cliff's delta T3 |
| --- | --- | --- | --- | --- |
| orange_etc | 0.01604339* | 0.1353941 | 0.90476191^#^ | 0.39583333 |
| Fruit | 0.01697033* | 0.05325674 | 0.85714286^#^ | 0.55833333^#^ |
| fresh_fish | 0.02029663* | 0.11792529 | 0.11792529 | 0.40833333 |
| tomatoes | 0.02769362* | 0.02769362* | 0.75396825^#^ | 0.44166667 |
| Fibre | 0.07547467 | 0.20284623 | 0.65079365^#^ | 0.34166667 |
| DHA | 0.07547467 | 0.68226962 | 0.65079365^#^ | 0.15 |
| pefruit | 0.07547467 | 0.08426602 | 0.64285714^#^ | 0.5125^#^ |
| avocado | 0.07547467 | 0.09311477 | 0.62698413^#^ | 0.47083333 |
| zucchini_etc | 0.08489524 | 0.05564111 | 0.5952381^#^ | 0.54166667^#^ |
| celery_etc | 0.09568192 | 0.05325674 | 0.05325674 | 0.63333333^#^ |
| yoghurt | 0.09568192 | 0.13936351 | 0.56349206^#^ | 0.39166667 |
| nuts...legumes | 0.10645757 | 0.09212805 | 0.55555556^#^ | 0.4875 |
| noodles | 0.09568192 | 0.74393346 | 0.55555556^#^ | 0.10416667 |
| apple_etc | 0.09568192 | 0.08426602 | 0.54761905^#^ | 0.5^#^ |
| spinach | 0.10965681 | 0.05325674 | 0.53174603^#^ | 0.60416667^#^ |
| carrots | 0.08489524 | 0.08426602 | 0.53174603^#^ | 0.4875 |
| muesli | 0.11502283 | 0.09311477 | 0.50793651^#^ | 0.4375 |
| peveg | 0.12778011 | 0.05171807 | 0.5^#^ | 0.69166667^#^ |
| pedairy | 0.13383055 | 0.3971291 | 0.49206349 | 0.25 |
| pePFA | 0.12778011 | 0.08560567 | 0.48412698 | 0.4625 |
| banana | 0.15728616 | 0.7171572 | 0.44444444 | 0.11666667 |
| EPA | 0.20513222 | 0.94589144 | 0.42857143 | 0.04166667 |
| pfpolyfats | 0.19882073 | 0.09212805 | 0.42857143 | 0.47916667 |
| dairy | 0.1972623 | 0.99221787 | 0.42857143 | 0.00833333 |

*Supplementary Table 5B: Foods associated with LMDA*

| Food/nutrient | pval trimester 2 | pval trimester 3 | Cliff's delta T2 | Cliff's delta T3 |
| --- | --- | --- | --- | --- |
| peaddedsugars | 0.02029663* | 0.05325674 | -0.8174603^#^ | -0.5708333^#^ |
| Sweet drinks | 0.02029663* | 0.05171807 | -0.8095238^#^ | -0.6791667^#^ |
| Disc snacks | 0.06317252 | 0.11662911 | -0.6904762^#^ | -0.4333333 |
| White bread | 0.04363903* | 0.58553128 | -0.5952381^#^ | -0.125 |
| Disc Meat | 0.21028606 | 0.05325674 | -0.4206349 | -0.5708333^#^ |
| milk | 0.1686758 | 0.05325674 | -0.4285714 | -0.5541667^#^ |
| Red meat | 0.11502283 | 0.16385877 | -0.531746^#^ | -0.3666667 |
| Disc. meals | 0.12598745 | 0.15037121 | -0.515873^#^ | -0.3791667 |
| Disc. dairy | 0.12758275 | 0.05325674 | -0.5079365^#^ | -0.5833333^#^ |
| pfSFA | 0.34605212 | 0.08560567 | -0.2777778 | -0.5^#^ |
| crumbed_chicken | 0.12598745 | 0.05325674 | -0.4920635 | -0.5458333^#^ |
| TFA | 0.77066436 | 0.09311477 | -0.1111111 | -0.475 |
| breakfast_cereal | 0.21094695 | 0.09311477 | -0.3888889 | -0.4625 |

For definitions please see Supplementary Table 1. Abbreviations: DHA, docosahexaenoic acid; Disc, discretionary; EPA, eicosapentaenoic acid; peaddedsugars, percentage of energy from added sugars; pePFA, percentage of energy from polyunsaturated fatty acids; pfpolyfats, percentage of fat from polyunsaturated fat; pfSFA, percentage of fats coming from saturated fatty acids.

*= pvalue <0.05; #= Cliff’s Delta value >5

Key:


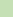
 Fruit and vegetables


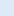
 Protein


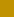
 Fibre


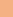
 Healthy fats


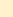
 Unhealthy fats


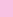
 Sugars


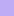
 Other processed food


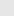
 Refined carbohydrates

Note that milk and breakfast cereals cannot be easily classified as the class would include both healthy and unhealthy dietary products, for example sweetened chocolate milk etc
